# Supplementary material for: Splice-Junction-Based Mapping of Alternative Isoforms in the Human Proteome
Source: Cell Rep. Author manuscript; Available in PMC 2020 Jan 15. (PMC6961840; doi:10.1016/j.celrep.2019.11.026)
Supplement: 2 [file NIHMS1546469-supplement-2.zip › DF1/PXD010154/Thyroid/Thyroid_16_ATXN3_EEGSLCAQHCLNNLLQGEYFSPVELSSIAHQLDEEER.pdf]

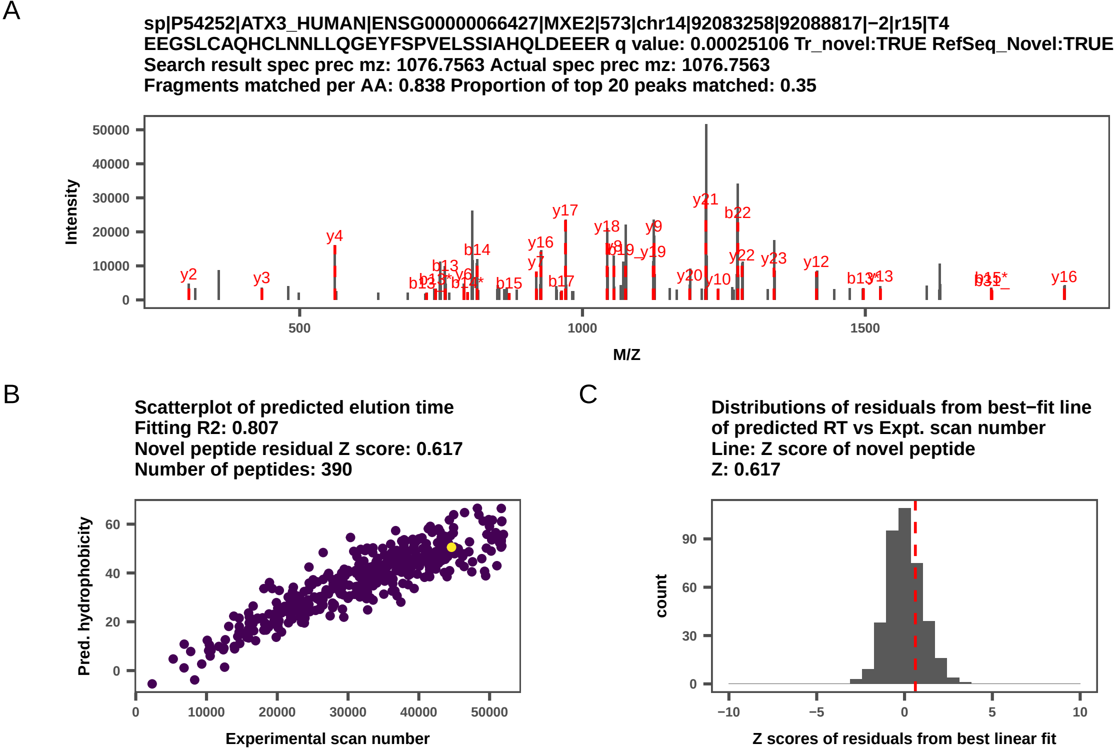
A
sp|P54252|ATX3_HUMAN|ENSG00000066427|MXE2|573|chr14|92083258|92088817|−2|r15|T4
EEGSLCAQHCLNNLLQGEYFSPVELSSIAHQLDEEER q value: 0.00025106 Tr_novel:TRUE RefSeq_Novel:TRUE
Search result spec prec mz: 1076.7563 Actual spec prec mz: 1076.7563
Fragments matched per AA: 0.838 Proportion of top 20 peaks matched: 0.35
Intensity
M/Z
B
Scatterplot of predicted elution time
Fitting R2: 0.807
Novel peptide residual Z score: 0.617
Number of peptides: 390
Pred. hydrophobicity
Experimental scan number
C
Distributions of residuals from best−fit line
of predicted RT vs Expt. scan number
Line: Z score of novel peptide
Z: 0.617
count
Z scores of residuals from best linear fit
